# Express Method of Preparation of Hollow Fiber Membrane Samples for Spinning Solution Optimization: Polysulfone as Example

**DOI:** 10.3390/membranes11060396

**Published:** 2021-05-27

**Authors:** Tatyana Anokhina, Alisa Raeva, Sergey Makaev, Ilya Borisov, Vladimir Vasilevsky, Alexey Volkov

**Affiliations:** A.V. Topchiev Institute of Petrochemical Synthesis, Russian Academy of Sciencs, 29 Leninsky Prospekt, 119991 Moscow, Russia; alisa0225@mail.ru (A.R.); makaev@ips.ac.ru (S.M.); boril@ips.ac.ru (I.B.); vasilevskii@ips.ac.ru (V.V.); avolkov@ips.ac.ru (A.V.)

**Keywords:** hollow fiber membranes, express method of preparation, spinning solution optimization, polysulfone, kinetic precipitation

## Abstract

This article describes a new technique for the preparation of hollow fiber (HF) membrane samples using an automatic manipulator unit. The manipulator uses a syringe needle to form a HF of a given geometry. The needle in automatic mode is sequentially immersed, first into the polymer solution and then into the coagulation bath. The possibility of using a manipulator to obtain HF samples was studied on the known polysulfone (PSf)/N-methylpyrrolidone (NMP)/pore-forming additive system. A series of HF membrane samples were made within 29 h from twelve 1 mL PSf casting solutions. This was 15 times faster than obtaining samples of HF membranes at the multifunctional research laboratory facility. From the point of view of the consumption of the components of the casting solution, the use of the manipulator was 30 times more economical, and the consumption of water for precipitation and washing was 8000 times less. The developed method made it possible to study samples of HF by scanning electron microscopy (SEM), ultrafiltration, and evaluate its mechanical properties without spinning the membranes. Using the new technique, the optimal composition of the casting solution for the wet spinning of HF PSf membranes was selected during two weeks. Thus, the manipulator makes it possible to significantly reduce the time of the new membrane preparation, reduce the volume of used polymer, and thus makes it promising to study expensive or new membrane materials.

## 1. Introduction

The successful implementation of membrane separation mainly depends on the membrane itself. In general, the structure and material of the membrane must correspond to the separation process for which the membrane was created. In membrane separation, both flat and hollow fiber (HF) membranes are used. HF polymer membranes are most often prepared from natural and synthetic polymers by the solution method using the phase inversion process [1]. The main advantage of HF membranes is the higher packing density of the membrane in the module (from 3000 to 20,000 m^2^/m^3^) compared to flat or tubular membranes [2,3,4], which significantly reduces the size of the separation devices. Phase inversion is a phase separation process where a polymer is transferred from a solution or melted state to a solid state in a controlled manner. There are several different approaches to the implementation of the phase inversion method [4,5]: the manufacture of HF membranes is carried out using the dry (evaporation induced phase inversion) or wet casting method (diffusion induced phase inversion) or a combination of them (dry-wet method) [6].

The initial solution, consisting of only one phase, breaks down into two: a solid polymer, which forms the matrix of the membrane, and a liquid, which forms pores in it. Due to contact with the non-solvent (coagulant), the upper surface quickly precipitates, forming a dense, selective layer. This layer slows down the penetration of the non-solvent agent into the polymer sublayer, which is deposited much more slowly and forms a more porous structure. The thermodynamic properties of the system and the kinetics of solvent and coagulant exchange have a strong influence on the morphology of the membrane, which in turn determines its transport and separation properties. Thus, for the manufacture of HF membranes with different transport and separation properties, it is important to select a polymer, a solvent, and a coagulant (non-solvent agent) for this [5,6]. However, the creation of polymer HF membranes depends not only on the choice of polymer, solvent, and non-solvent. The precipitation temperature, the time before precipitation, and the duration of the precipitation itself are all key parameters in the formation of HF membranes by phase inversion [7,8].

There are a large number of scientific papers on the influence of the conditions of HF formation on their membrane properties. The results of more than 160 scientific publications including studies on 23 industrial polymers were collected in the review by Feng et al. [9]. The 2019 review contains studies on the influence of the nature and composition of the internal coagulant, its temperature, and the contact time of the casting polymer solution with it [10]. The latest review of 2020 contains research on the development of casting methods of HF membranes that are safe for humans and the environment [11]

Polysulfone (PSf) is one of the most common polymers for the manufacture of HF membranes for micro-, ultrafiltration, gas separation, and membrane contactors [12,13,14]. In scientific works on the formation of HF membranes from this polymer, the influence of the molecular weight and concentration of the matrix polymer, the solvent used, the type of pore-forming agent, its molecular weight and concentration, the nature and composition of the coagulant on their transport and separation properties has been studied in detail [15,16,17,18].

All of the above works describe the production of HF polymer membranes on laboratory installations of dry-wet casting, which is associated with the production of a large amount of fiber, 200 m or more. The main problem is that to study the effect of casting conditions on the membrane properties of HF membranes, such a large amount is not necessary. In addition, the production of large volumes for research purposes entails large losses of reagents (polymer, pore-forming agent, solvent, and coagulant), and the time from the beginning of solution preparation to the formation of the membrane can take up to three days.

Another key factor in the preparation of polymer membranes using non-solvent induced phase separation is the determination of the membrane formation time, which is limited by the precipitation rate of the polymer solution. Usually, this is done by determining the diffusion coefficients of the coagulant in the “unlimited layer” of the polymer solution [19,20]. This approach enables us to determine the mechanism of transport of the insoluble agent into the polymer solution thickness for further phase decomposition and to qualitatively assess the effects of the polymer, its concentration, the selected solvent, and coagulant on the rate of solution precipitation. At the same time, it does not give an understanding of the real time of the formation of a polymer membrane of a given thickness and its final morphology.

Therefore, a method for determining the precipitation rate in a “limited layer” of a polymer solution was developed, which enables us to determine not only the precipitation time of a membrane with a given thickness, but also to predict the porous structure of the future membrane [21].

Thus, the purpose of this work was to select the composition of the casting solution and the conditions for HF membranes formation with external precipitation using new express methods for studying the properties of the polymer solution and polymer membranes.

## 2. Materials and Methods

### 2.1. Materials

In this work, polymer casting solutions and membranes were prepared from a commercial polymer-polysulfone (PSf) in BASF Ultrason^®^ S 6010 granules (Mw = 4.5–5.5 × 10^4^ g/mol, BASF, Ludwigshafen am Rhein, Germany). N-methylpyrrolidone (NMP) (Across Organics, Waltham, MA, USA) 99% extra pure was used as solvents. Pore-forming agents were introduced into the solution:

- polyvinylpyrrolidone (PVP) K 30 with a molecular weight of 40,000 g/mol produced by Sigma Aldrich Co. LLC, St. Louis, MO, USA, presented as a white powder; and
- polyethylene glycol (PEG400) in the form of a yellowish liquid with a molecular weight of 400 g/mol and a dynamic viscosity of 120 MPa s produced by Acros Organics, Waltham, MA, USA.

A model dye Blue Dextran with a molecular weight of 69,000 g/mol produced by Sigma Aldrich Co. LLC, St. Louis, MO, USA was used to evaluate the separation properties of the produced samples of HF membranes.

### 2.2. Preparation of Casting Solutions

Twelve casting polymer solutions of various compositions with a volume of 10 mL were prepared (Table 1). Solutions can be divided into three groups:

Group I: PSf-NMP;
Group II: PSf-NMP—5 wt% PVP; and
Group III: PSf-NMP—5 wt% PEG400.

In each group, PSf concentration varied from 15 to 24 wt%.

The components of the solution were mixed in a glass bottle at a temperature of 80 °C, then degassed and filtered for 24 h. A magnetic stirrer IKA C-MAG HS 10 was used for stirring.

### 2.3. Study of the Casting Solution

#### 2.3.1. Investigation of the Viscosity of Casting Solutions

A Brookfield DV III-Ultra rotary viscometer produced by Brookfield Engineering, (Middleborough, MA, USA) was used to measure the viscosity of the polymer solutions. Measurements for each of the solutions were carried out at a temperature of 25 °C. Three viscosity measurements were carried out for each polymer solution.

#### 2.3.2. Kinetics of Precipitation of Casting Solutions

To study the kinetics of the precipitation of PSf polymer solutions, the “limited” layer technique was used [21]. This enabled us to simulate the process of forming a polymer membrane of a given thickness and visualize the process of pore formation. By gluing two cover glasses with double-sided tape, a rectangular channel with a depth (*d*) of 300–400 µm was formed, open to the atmosphere on one side. The channel was then filled with a PSf solution. The sample with a polymer solution was fixed on a slide. Using a Pasteur pipette, a coagulant (water) was added into the polymer solution from the side open to the atmosphere, and the process of phase separation of the PSf solution was observed using a microscope and recorded on a video camera.

The kinetics of polymer precipitation was evaluated using the precipitation rate of a polymer solution in a layer of a given thickness. This was calculated as the ratio of the total thickness of the polymer layer (*d*, µm) to the time of its precipitation (*t*, s).

(1) $$v_{l}=\frac{d}{t}$$

The precipitation rate was averaged based on five measurements for each polymer solution.

### 2.4. Preparation of Short-Samples of Hollow Fiber Membranes

**Figure 1:**
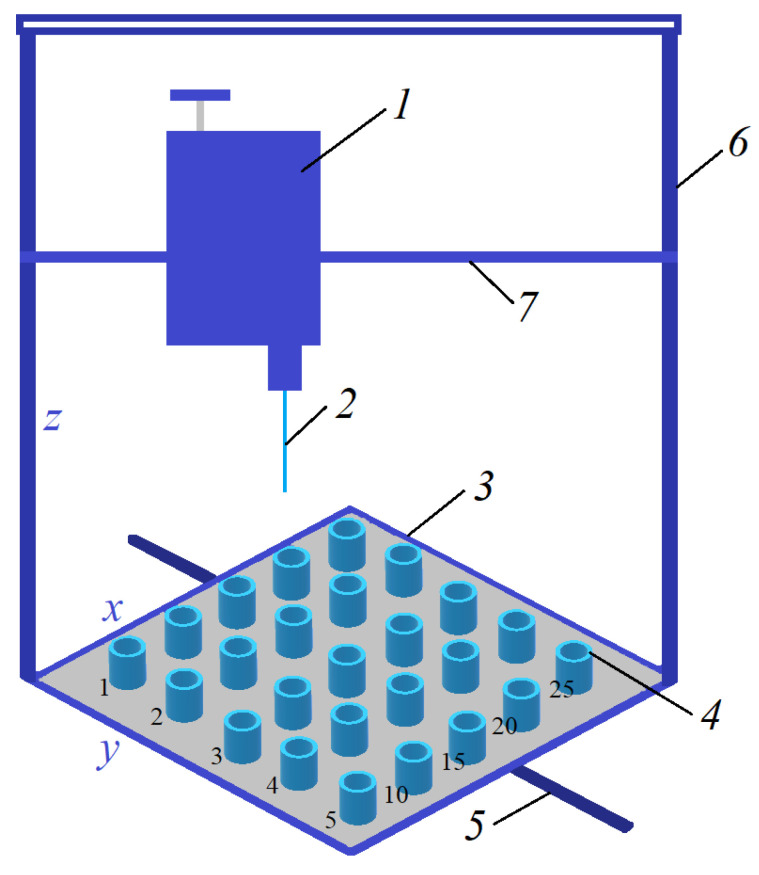
The manipulator created in the work for the production of short-samples of hollow fiber (HF) polysulfone (PSf) membranes.

To prepare short-samples of HF membranes, a new original manipulator based on a 3D printer was developed, which enabled us to prepare up to 25 short-samples of HF membranes (Figure 1).

The manipulator (Figure 1) includes a block (1) with a carrier needle (2) moving along the vertical (6) and horizontal (7) bars in the x and z axes. Short-samples of HF membranes were formed by successively lowering the carrier needle into sample bottles (4) with a casting solution, and then into sample bottles (4) with a coagulant. The sample bottles with casting solutions and coagulants are located on a platform (3), which also moves along the bar (5) in y axis. Short-samples of HF membranes were prepared for the study of morphology, mechanical, transport, and separation properties.

For the manipulator, software has been developed that enables one to set the algorithm for the movement of the carrier needle and the platform with sample bottles to prepare 25 short-samples of HF membranes. The developed program of the manipulator enabled us to set the speeds of movement of the carrier needle in axes x (horizontally along the polymer platform with bottles) and z (movement of the carrying needle perpendicular to the polymer platform) and of the polymer platform with bottles in axis y (vertical movement of the platform) as well as the contact time of the carrier needle with the casting solution and the coagulant. To obtain each short-sample of the HF PSf membrane, an algorithm for the movement of the carrier needle and platform with bottles was created, consisting of the following commands:

1. home—placing the unit with the carrier needle and the platform with the weighing bottles in the starting position.
2. move—movement to the bottle with polymer solution. The speed of movement along axes X, Y is 0–50 mm/s.
3. moveZ—lowering the unit with the carrier needle into a weighing bottle with a polymer solution. The speed of movement along the Z axis is 0–20 mm/z.
4. dwell—holding the carrier needle in a bottle with a polymer solution to form a polymer solution on it. The holding time of the carrier needle is 20 s.
5. moveZ—lifting the carrier needle with polymer solution. The speed of movement along the Z axis is 0–20 mm/s.
6. dwell—holding the carrier needle over the bottle. On average, one sample requires 46 s.
7. move—movement of the carrier needle with the polymer solution to the weighing bottle with the precipitant. The speed of movement along the X, Y axes is 0–50 mm/s.
8. moveZ—lowering the carrier needle with the polymer solution into the weighing bottle with the precipitant. The speed of movement along the Z axis is 0–20 mm/s
9. dwell—holding the carrier needle in a weighing bottle with a precipitant. On average, one sample requires 33 s.
10. moveZ—raising the carrier needle to a starting height of 0 mm. The speed of movement along the Z axis is 0–20 mm/s.
11. home—return to the starting position to replace the carrier needle from the membrane with a new one. The speed of movement along the X, Y axes is 0–50 mm/s.

Using a carrier needle, a wet method of forming HF membranes was carried out. The speed of the carrier needle movement along the x, y, z axes was selected relative to the viscosity of the casting solution. Distilled water was used as a coagulant. The contact time of the needle (carrier of the polymer solution) with the coagulant corresponded to the time of precipitation of this solution in the “limited” layer. After precipitation, the samples were washed alternately in two bottles with water. During one cycle of operation of the manipulator, 12 short-samples of HF membranes were formed. The maximum fiber length was 75 mm.

### 2.5. Investigation of the Properties of Short-Samples of Hollow Fiber Membranes

#### 2.5.1. Study of the Morphology of Short-Samples of HF PSf Membranes

Scanning electron microscopy (SEM) was used to characterize the structure and morphology of the membranes. SEM was carried out on a Thermo Fisher Phenom XL G2 Desktop SEM, Waltham, MA, USA. Cross-sections of the membranes were obtained in liquid nitrogen after preliminary impregnation of the specimens in isopropanol. A thin (5–10 nm) gold layer was deposited on the prepared samples in a vacuum chamber (~0.01 mbar) using a desktop magnetron sputter “Cressington 108 auto Sputter Coater”, (Rassendale, Liverpool, UK). The accelerating voltage during image acquisition was 15 keV. Further image analysis and determination of the selective layer thickness was carried out using the Gwyddion software (ver. 2.53 Brno, Czech Republic).

#### 2.5.2. Investigation of Mechanical Properties of Short-Samples of HF PSF Membranes

Mechanical characteristics were studied using a TT-1100 bursting machine (Cheminstruments, Fairfield, CT, USA) at room temperature (22–24 °C). The traverse travel speed was 3.8 cm/min. The samples possessed a length of 70 mm. The initial distance between the clamps was 50–60 mm. Young modulus was determined by the slope of the initial stress-strain dependence close to the rectilinear section at strain values not exceeding 5%. The stresses were calculated on the initial cross-section of the sample.

#### 2.5.3. Investigation of Transport and Separation Properties of Short-Samples of Hollow Fiber PSF Membranes

**Figure 2:**
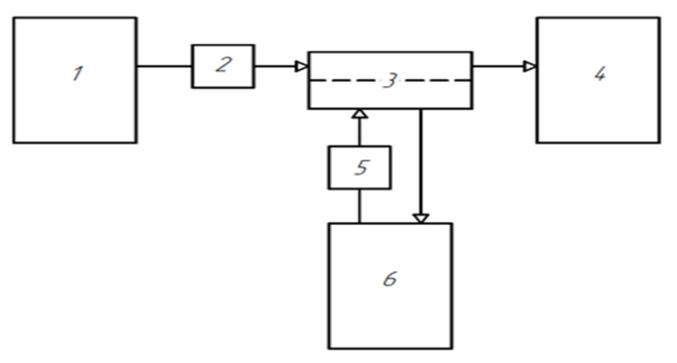
Diagram of the installation for measuring water permeability and the rejection. 1—the storage tank with the initial solution; 2—the ultrafiltration pump; 3—the ultrafiltration module; 4—the retentate and regenerate storage tanks; 5—the regeneration pump; 6—the storage tank for the filtrate (permeate).

The study of the permeability of the prepared short-samples was carried out in an installation as shown in Figure 2. The short-sample was sealed into the module. The measurement was performed in the flow mode with a transmembrane pressure of 1 atm.

The permeability was calculated using the formula:

(2) $$P=\frac{V}{S\cdot t\cdot \Delta p}$$

where *P* is the permeability (L/m^2^ h bar); *V* is the volume of the taken sample (l); *t* is the sampling time (h); *S* is the surface area of the selective layer of the short-sample of the hollow fiber (m^2^); and Δ*p* is the overpressure (bar).

The calculation of the rejection rate was conducted with the formula:

(3) $$R=\frac{1-C_{p}}{C_{f}}\cdot 100$$

where *R* is the rejection (%); *C_p_* is the concentration of the solute in the permeate (mg/L); and *C_f_* is the concentration of the solute in the feed stream (mg/L).

To measure the rejection, an aqueous solution of Blue Dextran (MM = 69 kg/mol) with a concentration of 100 mg/kg was prepared and used.

## 3. Results and Discussion

### 3.1. Viscosity and Precipitation Rate of Polymer Casting Solutions

**Figure 3:**
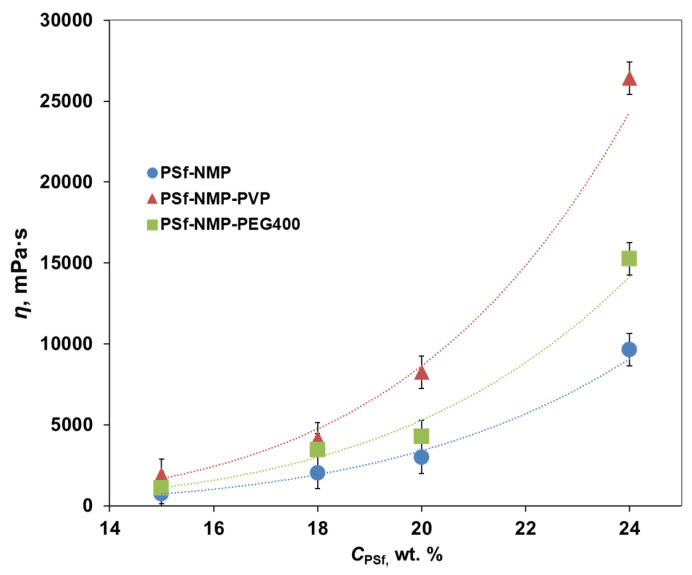
Dynamic viscosity of PSf in N-methylpyrrolidone (NMP) casting solutions. I—PSf-NMP; II—PSf-NMP-PVP; III—PSf-NMP-PEG.

In order to predict the properties of future hollow fiber PSf membranes and to select the casting conditions, the first stage of the work was carried out to study the properties of all prepared casting solutions. Their dynamic viscosity was measured (Figure 3).

As PSf concentration increased from 15 wt% to 24 wt%, dynamic viscosity increased monotonically (Figure 3). For PSf solutions in NMP without pore-forming agents (group I), the viscosity increased from 740 to 9630 MPa s; for solutions with 5 wt% PVP (group II) from 1870 to 26,420 MPa s; and for solutions with 5 wt% PEG400 (group III) from 1110 to 15,250 MPa s. At the same time, it can be seen from Figure 3 that solutions containing pore-forming agents had a higher dynamic viscosity than solutions without them, which corresponds to the literature data [15,22,23]. For each PSf concentration, the viscosity of solutions with 5 wt% PEG400 was about 1.5 times higher, and with 5 wt% PVP, it was 2.5–3 times higher than the viscosity of PSf solutions without pore-forming agents. The greatest effect of the introduction of the pore-forming agent on the viscosity was observed at a high concentration of PSf (24 wt%). The viscosity of solutions with the addition of pore-forming agents, which were also polymers, increased due to an increase in the total polymer content [22]. In group II solutions, this effect was enhanced as the introduction of PVP into PSf-NMP solution led to thermodynamic instability [24].

In addition to the dynamic viscosity, the precipitation rates for all prepared casting solutions were investigated. The precipitation rate was studied using the “limited” layer method, which most accurately simulates the process of forming the wall of a HF when it is prepared [21].

**Figure 4:**
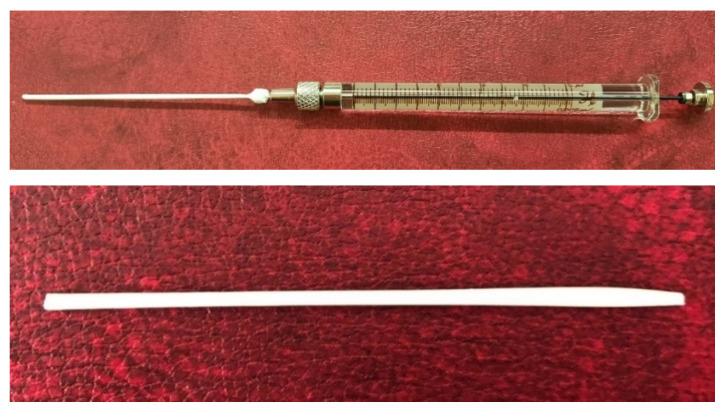
An example of the resulting short-sample of a HF PSf membrane.

The precipitation rate of the solutions as well as the viscosity depends on the concentration of PSf (Table 2). Comparing Figure 3 and Figure 4, the correlation of these two parameters of the casting solution was observed. In this case, the precipitation rate of the casting solution was inversely proportional to the viscosity [20], since it decreased as PSf concentration increased. For group I solutions with an increase in PSf concentration from 15 to 24 wt%, the precipitation rate decreased from 24.5 to 6 µm/s; for group II from 14.8 to 4.2 µm/s; and for group III from 21.0 to 7.2 µm/s. Note that this decrease cannot be regarded as monotonically decreasing. In addition to dividing the solutions into groups from the point of view of the introduced pore-forming additive, they can be divided into low-concentration (*C*_PSf_ = 15–18 wt%) and high-concentration (*C*_PSf_ = 20–24 wt%). Analyzing the obtained values of the deposition rates for all solutions, it can be concluded that for PSf-NMP solutions on going from 15 to 18 wt% and from 20 to 24 wt%, the deposition rate decreased by 28%, for PSf-NMP-PVP solutions by 25%, and for PSf-NMP-PEG400 solutions by 15%. However, when going from low-concentration to high-concentration solutions, there was a sharp decrease in the deposition rate by about 60% in all groups. This effect is possibly associated with an increase in the viscosity of solutions during PSf concentration, which exhibits an exponential character (Figure 3).

The precipitation rates in groups I, II, and III of the polymer solutions were compared. Table 2 shows that the precipitation rates of PSf solutions in NMP without pore-forming agents and with the addition of 5 wt% PEG400 were comparable, despite the fact that the viscosities of group I solutions were about 1.5 times higher than for group III solutions. This may be due to the fact that PEG400 is not only a pore-forming agent, but is also a non-solvent in relation to the PSf-NMP polymer solution. The introduction of a non-solvent into the polymer solution in small amounts increases the precipitation rate [19]. The hydrophilicity of PEG400 also increases the inflow rate of water diffusion in the polymer solution [25]. Thus, the combination of properties of PEG400 and its low content compensates for the effect of viscosity on the precipitation rate of polymer solutions, so they are comparable for groups I and III.

The precipitation rate of group II of PSf solutions with 5 wt% of the pore-forming agent PVP was 1.5–1.7 times lower than for solutions of groups I and III. The greatest difference between the precipitation rates of solutions of groups I and II was observed at low concentrations of PSf, up to 15 wt%, and the difference in precipitation rate decreased at higher concentrations. This was also due to thermodynamic instability, which decreased as the ratio between PSf and the PVP additive decreased from 25 to 17 wt% [23].

### 3.2. The Porous Structure of Short-Samples of Hollow Fiber Membranes Formed on the Needle Carrier of the Manipulator

Next, short-samples of HF PSf membranes were prepared on a carrier needle from each casting solution using our unique manipulator created for this work. Using a carrier needle, a wet method of forming HF membranes was carried out.

First, in this work, a “wet” method of forming membranes was performed using a manipulator. Basically, the “wet” method of implementing the process of phase decomposition of a polymer solution in contact with a non-solvent is carried out to obtain flat asymmetric membranes [26]. However, there are known works in which the “wet” method has been used to obtain membranes in the form of a hollow fiber [22,27,28]. During molding, the HF from the die immediately dropped into the sedimentation bath. Water was used as a precipitator to induce instantaneous phase decay from the outside of the fiber [28]. Bore fluid is present in the manufacturing process of the HF membrane. It is necessary for the formation of an internal channel, and not for the implementation of the phase decomposition of the polymer solution from the inside of the fiber. SEM photos of HF membranes indicate the movement of the precipitator front from the outer surface of the HF to the inner, and not vice versa, according to [27,28]. To achieve this effect, a composition of body fluid was selected to exclude the possibility of phase decomposition of the polymer solution from the inside, and the deposition front was from the outside. Thus, the polymer solvents were NMP and DMF with a small addition of water from 1 wt% [28] to 10 wt% [27] was used as bore fluid.

The created manipulator enabled the simulation of the process of the phase decomposition of a polymer solution from the outside, and the carrier needle played the role of bore fluid for the formation of an internal channel.

Different values of the speed of movement of the carrier needle and the residence time in the precipitator were set in the manipulator program due to the difference in the physicochemical properties of the molding solutions (Table 3). For all membrane samples, the speed of lowering the carrier needle along the z axis into the weighing bottle with a polymer solution and a precipitant was 20 mm/s. The speed of movement of the carrier needle and the polymer platform along the X, Y axes was 50 mm/s.

The holding time of the carrier needle in the bottle with the polymer solution was increased due to the increase in the viscosity of the solutions, while the lifting rate of the carrier needle, in contrast, decreased (Table 3).

Short-samples of HF membranes were prepared to study their morphology, mechanical, transport, and separation properties. During one cycle of the manipulator operation, 12 short-samples of HF PSf membranes were prepared. An example of the resulting short-sample of a HF PSf membrane on a carrier needle is shown in Figure 4.

The effect of the composition of the casting solution on the morphology of the prepared short-samples of HF PSf membranes was studied using a scanning electron microscope (SEM) (Table 4). In group I of the polymer solutions, the number of finger-shaped transport channels and diameter decreased, and the proportion of spongy pores increased with an increase in PSf concentration from 15 to 24 wt%. In group II, with an increase in the concentration of PSf, the formation of finger-shaped transport channels of a smaller diameter was observed, while the number of these channels increased. In group III solutions (with the addition of 5 wt% PEG400), there was a transition from a structure with finger-shaped transport channels at 15 wt% PSf to a dense structure with spongy pores in the case of 24 wt% PSf. At the same time, the presence of spongy pores already began to prevail with a PSf concentration of 18 wt%. Thus, PSf-NMP and PSf-NMP-5 wt% PEG400 solutions formed a denser structure as the polymer concentration increased. For solutions with 5 wt% PVP, in contrast, the most developed structure with transport channels was formed.

As shown in Table 2 and Table 3 for solution groups I and III, the precipitation rate and precipitation time were comparable. In group II, the precipitation time was almost two times longer.

In order to explain the data obtained from the precipitation rate and time, the Hansen solubility parameters were analyzed for the pore-forming agents, NMP solvent, and water coagulant (Table 5).

Table 5 shows that the solubility parameters responsible for the polarity (*δ*_p_) and for the formation of hydrogen bonds (*δ*_h_) were comparable for PVP (*δ*_p_ = 11.7 MPa^1/2^; *δ*_h_ = 8.6 MPa^1/2^) and NMP (*δ*_p_ = 12.3 MPa^1/2^; *δ*_h_ = 7.2 MPa^1/2^). Therefore, the dissolution of PVP in the coagulant (water) took longer than the dissolution of PEG400, which was why the precipitation process itself took longer in the group of polymer solutions with PVP.

The SEM method showed that in the absence of a pore-forming agent, large spongy pores were formed (PSF-20 and PSf-24 membranes). Introduction of 5 wt% PEG400 in solutions with the same concentration led to a decrease in the diameter of the spongy pores. At the same time, at a PSf concentration of 24 wt%, a denser near-surface selective layer with a thickness of about 30 µm was formed in the membrane. Addition of 5 wt% PVP in PSf-NMP solutions led to the preparation of a membrane with the densest support layer.

To date, the use of a carrier needle does not enable one to control the wall thickness of the resulting HF membrane. The lack of alignment in the HFs is presumably related to some deviation of the carrier needle from an angle of 90°. At the same time, this is an advantage from the point of view of studying the evolution of the porous structure of the membrane along its wall thickness. Thus, for example, in PSF-20 and PSf-PVP-24 membranes, when the wall thickness increased from 200 to 350 µm and from 220 to 400 µm, respectively, macrovoids began to form in the support sponge layer. In PSF-PEG-18 and PSf-PEG-20 membranes prepared from group II solutions, finger-shaped pores formed along the entire wall at a thickness of about 50 µm. However, with an increase in the wall thickness in these membranes, the layer with finger-shaped pores was replaced by a dense support layer with spongy pores. In addition, the wall thickness increased with an increase in the viscosity of the solution [18].

### 3.3. Mechanical Properties of Short-Samples of Hollow Fiber Membranes

**Figure 5:**
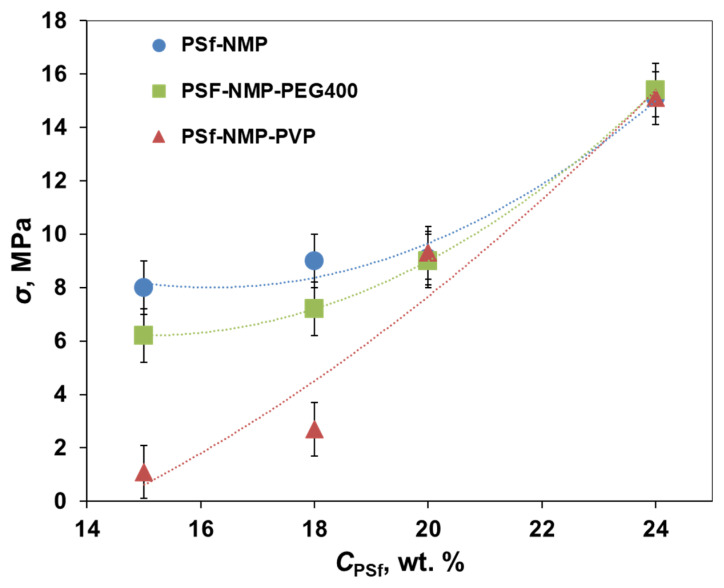
Mechanical properties of short-samples of hollow fiber PSf membranes.

The influence of the composition of the casting solution for the preparation of short-samples of hollow fiber PSf membranes using the manipulator was studied from the point of view of the mechanical properties (Figure 5).

Figure 5 shows an increase in the tensile strength of membranes with an increase in the concentration of polymer in the casting solution. For membranes prepared from group I solutions, it increased from 8 to 15.1 MPa; from group II from 1.1 to 15.1 MPa; and from group III from 6.2 to 15.4 MPa. Addition of 5 wt% of pore-forming agents in PSf solutions with concentrations of 15 and 18 wt% led to a decrease in the strength of the membranes, especially when using PVP. For PEG400, in the case of 15 wt%, the strength of the membranes decreased from 8 to 6.2 MPa, and in the case of 18 wt% from 9 to 7.2 MPa. For PVP in the case of 15 wt%, the strength of membranes decreased from 8 to 1.1 MPa, and in the case of 18 wt% from 9 to 2.7 MPa. Such a drop in strength with the addition of PVP may be due to the formation of macrovoids in the support layer of PSf-PVP-15 and PSf-PVP-18 membranes, as shown in the SEM photos (Table 4). The introduction of pore-forming agents into highly concentrated PSf solutions did not lead to a change in the mechanical properties of HF membranes. This may be explained either by the formation of a spongy structure in the walls of PSf HF membranes PSf-20, PSf-24, PSf-PEG400-20, PSf-PEGG400-24, or by the formation of thick walls due to the high viscosity of the casting solutions, as in the case of the PSF-PVP-20 and PSf-PVP-24 membranes.

### 3.4. Transport and Separation Properties of Short-Samples of Hollow Fiber PSF Membranes

All short-samples of hollow fiber PSf membranes were examined in terms of permeability (Table 6) and the rejection of the model dye Blue Dextran with a molecular weight of 69 kg/mol.

Table 5 shows a decrease in the permeability of membranes with an increase in the concentration of polymer in the casting solution. For membranes prepared from group I solutions, it decreased from 17.4 to 0.5 L/m^2^ h bar; from group II from 178.7 to 35 L/m^2^ h bar; and from group III from 475.6 to 0.1 L/m^2^ h bar. At the same time, introduction of 5 wt% PEG400 significantly increased the permeability of PSF-PEG400-15 and PSf-PEG400-18 membranes compared to the PSF-15 and PSf-18 membranes. The latter were made from similar solutions, but without the addition of a pore-forming agent. When PSf concentration increased to 20 wt%, there was a sharp drop in the water permeability in group III solutions to 28.6 L/m^2^ h bar in the PSf-PEG400-20 membrane. With a further increase in PSf concentration to 24 wt%, the formation of a near-surface dense layer with a thickness of about 30 µm (Table 4) reduced the permeability of the PSf-PEG400-24 membrane by two orders of magnitude.

Addition of 5 wt% PVP in PSF-NMP casting solutions kept the permeability of the membranes high across the whole studied range of PSf concentrations compared to other membranes prepared from group I and III solutions.

All short-samples of HF PSf membranes, except for PSf-PEG400-15, had a high rejection of 99.9%. The rejection (*R*, %) of PSf-PEG400-15 was 87.8%. The reduced *R* and high *P* for this membrane relative to the others seem to be due to the high ratio between PSf and PEG400, equal to 25%, which led to a less dense structure with finger-shaped pores.

### 3.5. Analysis of Economic and Time Costs

Since a new manipulator was developed, the first work was especially carried out on a well-known and widely studied system: the PSf/NMP/pore-forming additive. This system is used to produce HF membranes by the dry-wet method [15,16,17,31,32,33,34,35]. Traditionally, the selection of the optimal composition of the polymer solution is carried out by the multifunctional research laboratory facility. It consists of a large number of structural elements: a pressure reducing valve over the bore fluid, pressure reducing valve over the dope solution, tank with the bore fluid, tank with the spinning dope, thermostat for heating the bore fluid, spinneret block, first coagulation (spinneret) bath, second coagulation (washing) bath, rinsing and winding bath, system of guide rollers, and take-up drum [36]. The installation must go to a stationary spinning mode to obtain defect-free HF membranes. Therefore, the polymer solution must have a volume of at least 300 mL, the mixing time of which takes 24 h. Then, the solution is degassed and filtered. This process takes at least 24 h depending on the composition and viscosity of the solution [37]. When using the multifunctional research laboratory facility, the membrane fabrication time takes from 30 min. The fibers formed during the first and last five minutes are discarded and not used for research. Thus, 200 m of an experimental sample of a HF PSF membrane is obtained from 300 mL of a polymer solution. However, the study takes a maximum of 0.6 m (50 mm used for the SEM study, 50 mm for the study of mechanical properties, and 75–500 mm for the study of transport and separation characteristics). About 70 g of polymer is needed to prepare 24 wt% PSf solution. The remaining 230 g is a solvent with a pore-forming additive. Furthermore, the laboratory facility includes three sequential baths for precipitation and washing with a volume of 80 L [37]. Thus, the traditional method of obtaining HF PSF membranes includes not only a large consumption of the components of the spinning solution, but also distilled water.

A total of 10 mL of a solution of each composition was prepared for the preparation of short-samples of HF PSF membranes on a manipulator. This amount of solution was necessary to carry out three measurements of viscosity, five measurements of the deposition rate, and obtain nine samples of membranes for studies of the porous structure, mechanical properties, and deposition rate. One short-sample requires less than 1 mL of solution. The volume of the aqueous precipitation bath was 10 mL. The preparation time for each solution, together with degassing and filtration, took 24 h. Due to the small volume, 12 solutions were prepared simultaneously. About 3 min is necessary to obtain one short-sample taking into account the choice of the maximum speed of the carrier needle and the average holding time.

The study of the well-known molding composition (PSF-NMP-pore-forming additive) made it possible to calculate and compare the material and time costs for the manufacture of samples of HF PSf membranes on the manipulator and at the laboratory facility. The calculation of the cost of the components of the mixture for both methods of production is presented in Table 7. Information on the cost of the components of the mixture was searched for on the website of the suppliers. Component consumption was determined based on the volumes of molding solutions: 300 mL ≈ 300 g for the laboratory facility, 10 mL ≈ 10 g for the manipulator.

Analysis of the consumption and cost of the mixture components required for the preparation of 12 molding solutions demonstrate that the use of a manipulator to obtain research samples of HF membranes was 30 times economically more profitable than the laboratory facility. At the same time, the calculation did not take into account the consumption of distilled water and the energy consumption for its production for 12 solutions: the precipitation and washing bath of the laboratory facility of 2880 L vs. precipitation and washing bath manipulator of 360 mL.

Table 8 shows the calculation of the sample preparation time at the laboratory facility and at the manipulator for 12 solutions.

The calculation of the time costs showed that the manipulator reduced the time to obtain research samples of HF membranes from 12 spinning solutions by almost 15 times.

## 4. Conclusions

In order to analyze the influence of the composition of the casting solution on the morphology, mechanical, transport, and separation properties of the HF membranes, a new unique manipulator based on a 3D printer was designed. The manipulator used a syringe needle to form a HF of a given geometry. The needle in automatic mode was sequentially immersed first into the polymer solution and then into the coagulation bath. Special software was developed for the manipulator, which enabled us to obtain the speed of the carrier needle along the x, y, z axes and the platform with containers for various purposes (casting solutions, coagulants, etc.) as well as the residence time of the carrier needle within the casting solution and the coagulant. During one cycle of operation of the manipulator, 12 short-samples of HF membranes formed from solutions of various compositions.

The possibility of using a manipulator to obtain HF samples was studied on the known PSf/NMP/pore-forming additive system. A series of HF membrane samples were made within 29 h from twelve 1 mL PSf casting solutions. This was 15 times faster than obtaining samples of HF membranes at the multifunctional research laboratory facility. From the point of view of the consumption of the components of the casting solution, the use of the manipulator was 30 times more economical, and the consumption of water for precipitation and washing was 8000 times less

Having analyzed short-samples of HF PSf membranes, it was found that the best transport and separation characteristics were demonstrated by the membranes prepared from solutions with the addition of 5 wt% PVP with a PSf content of 15 to 24 wt%. The permeability of the membranes varied from 178.7 to 35 L/m^2^ h bar with the rejection of the Blue Dextran model dye (MM = 69 kg/mol) of 99.9% over the entire range of PSf concentrations in the casting solution. Moreover, these membranes demonstrated higher strength (9 and 15 MPa, for 20 and 24 wt% PSf in NMP with 5 wt% PVP, respectively) compared to the other samples. Taking into account the combination of morphology, mechanical, transport, and separation properties of short samples of HF membranes, casting solutions of 20 and 24 wt% PSF in NMP with the addition of 5 wt% PVP pore former are the most suitable compositions.

Thus, the new manipulator makes it possible to significantly reduce the time of the technological process of polymer membrane preparation and the volume of used polymer, which makes it promising to study expensive or previously unknown new membrane materials.

## Figures and Tables

**Table 1:** Compositions of the studied casting solutions.

No. Groups	No. Membrane	Polymer	*C*_pol_, (%)	Solvent	*C*_sol_, (%)	Pore-Forming Agent	*C*_agent_, (%)
I	PSf-15	PSf	15	NMP	85	-	-
	PSf-18		18		82		
	PSf-20		20		80		
	PSf-24		24		76		
II	PSf-PVP-15		15		80	PVP	5
	PSf-PVP-18		18		77		
	PSf-PVP-20		20		75		
	PSf-PVP-24		24		71		
III	PSf-PEG-15		15		80	PEG400	5
	PSf-PEG-18		18		77		
	PSf-PEG-20		20		75		
	PSf-PEG-24		24		71		

**Table 2:** The precipitation rate of PSf in NMP casting solutions. I—PSf-NMP; II—PSf-NMP-PVP; III—PSf-NMP-PEG.

*v*, (μm/s)			
*C*_pol_, (%)	PSf-NMP	PSf-NMP-PVP	PSf-NMP-PEG400
15	24.5 ± 0.6	14.8 ± 0.7	20.9 ± 0.5
18	18.7 ± 0.5	10.5 ± 0.5	18.3 ± 0.5
20	8.7 ± 0.2	5.5 ± 0.3	8.7 ± 0.2
24	6.0 ± 0.1	4.2 ± 0.2	7.2 ± 0.2

**Table 3:** Forming parameters for short-samples of HF PSf membranes.

Membrane No.	*H* (mPa s)	*t*_pr_ (s)	*t_hs_* (s)	*v_lifting_* (mm/s)	*t_hp_* (s)
PSf-15	730	11	0	20	11
PSf-18	2040	15	30	15	15
PSf-20	2980	38	40	15	38
PSf-24	9630	41	60	10	41
PSf-PVP-15	1870	20	30	15	20
PSf-PVP-18	4130	24	60	14	24
PSf-PVP-20	8260	61	70	12	61
PSf-PVP-24	26,420	76	73	10	76
PSf-PEG-15	1110	15	0	15	15
PSf-PEG-18	3470	18	60	15	18
PSf-PEG-20	4270	36	60	14	36
PSf-PEG-24	15,260	40	70	12	40

*t*_pr_—time precipitation of the polymer solution; *t_hs_*—time of holding of the Carrier Needle over a solution bottle; *v_lifting_*—velocity of lifting the carrier needle along the axis z; *t_hp_*—time of holding of the Carrier Needle over a bottle with precipitant.

**Table 4:** Scanning electron microscopy (SEM) photographs of hollow fiber membranes made from polymer solutions. I—PSf-NMP; II—PSf-NMP-PVP; III—PSf-NMP-PEG.

Group Solution	Membrane No.	SEM	
I	PSf-15	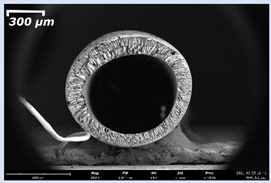	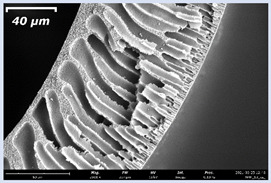
	PSf-18	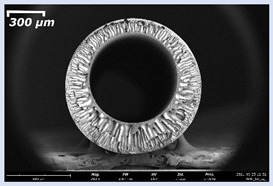	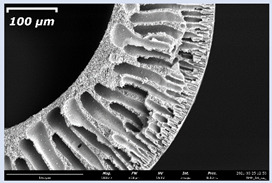
	PSf-20	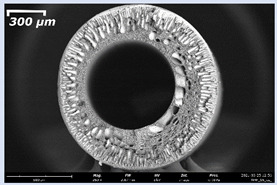	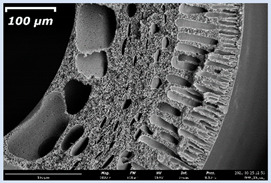
	PSf-24	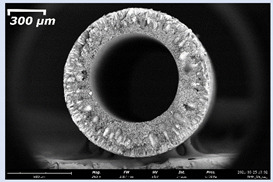	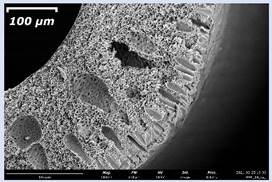
II	PSf-PVP-15	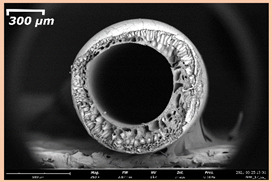	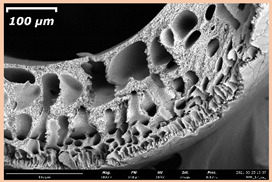
	PSf-PVP-18	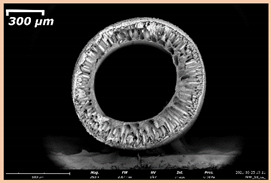	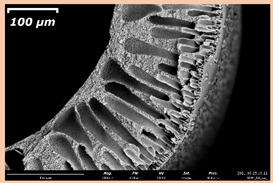
	PSf-PVP-20	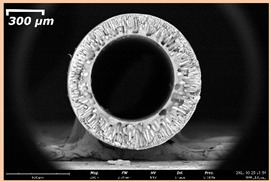	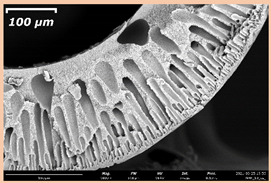
	PSf-PVP-24	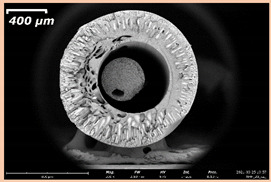	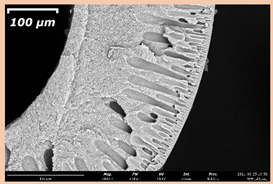
III	PSf-PEG-15	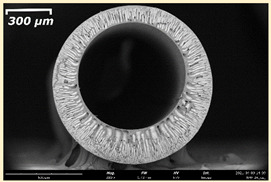	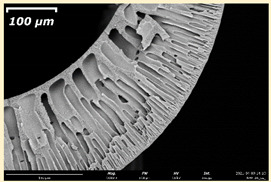
	PSf-PEG-18	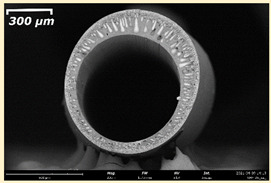	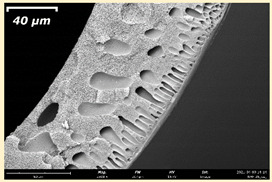
	PSf-PEG-20	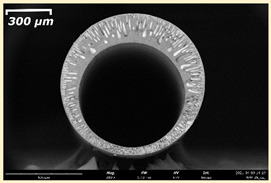	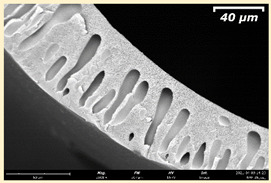
	PSf-PEG-24	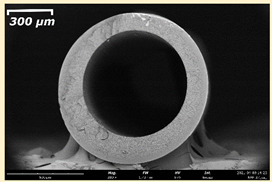	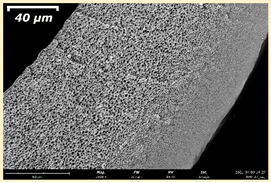

**Table 5:** Solubility parameters of the pore-forming agents, solvent, and coagulant.

	*δ*_d_ (MPa)^1/2^	*δ*_p_ (MPa)^1/2^	*δ*_h_ (MPa)^1/2^	*δ*_t_ (MPa)^1/2^	Reference
PEG400	16.6	3.7	13.3	21.6	[29]
PVP	15.5	11.7	8.6	21.2	[22]
NMP	18.0	12.3	7.2	22.9	[30]
Water	15.6	16.0	42.3	47.8	

*δ*_d_—the contribution of the dispersion interaction; *δ*_p_—the contribution of the polar interaction; *δ*_h_—the contribution of the interaction of hydrogen bonds; *δ*_t_—Hansen solubility parameter.

**Table 6:** Water permeability (P, L/m^2^ h bar) of short-samples of hollow fiber PSf membranes.

P (L/m^2^ h Bar)			
*C*_p_, (%)	No Additive	PVP	PEG400
15	17.1	178.7	475.6
18	5.7	94.3	89.7
20	2.9	82.4	28.6
24	0.5	35.0	0.1

**Table 7:** Calculation of the cost of the components of 12 molding solutions for the manufacture of samples at the laboratory facility and on the manipulator.

Groups	*C*_PSf_ (%)	Added	*C*_add_ (%)	*C*_NMP_ (%)	Laboratory Facility		Manipulator	
					Consumption (PSf/Add/NMP) (g)	Cost ($)	Consumption (PSf/Add/NMP) (g)	Cost ($)
I	15	-	-	85	45/-/255	81	1.5/-/8.5	2.7
	18			82	54/-/246	88	1.8/-/8.2	2.9
	20			80	60/-/240	92	2.0/-/8.0	3.1
	24			76	72/-/228	111	2.4/-/7.6	3.4
II	15	PVP	5	80	45/15/240	106	1.5/0.5/8.0	3.5
	18			77	54/15/231	118	1.8/0.5/7.7	3.9
	20			75	60/15/225	126	2.0/0.5/7.5	4.2
	24			71	72/15/213	142	2.4/0.5/7.1	4.7
III	15	PEG400	5	80	45/15/240	81	1.5/0.5/8.0	2.7
	18			77	54/15/231	89	1.8/0.5/7.7	3.0
	20			75	60/15/225	94	2.0/0.5/7.5	3.1
	24			71	72/15/213	103	2.4/0.5/7.1	3.4
Amount $:						≈1230		≈41

The price of 1 g PSf was ≈ 0.9 $, of 1 g NMP ≈ 0.16 $, of 1 g PVP K30 ≈ 0.6 $, of 1 g PEG400 ≈ 0.06 $.I—PSf-NMP; II—PSf-NMP-PVP; III—PSf-NMP-PEG.

**Table 8:** Calculation of the sample preparation time at the laboratory facility and at the manipulator for 12 solutions.

Groups	*C*_PSf_ (%)	Added	Laboratory Facility			Manipulator		
			*t*_mix_ (h)	*t*_degas/filtration_ (h)	*t*_spinning_ (h)	*t*_mix/degas_ (h)	*t*_filtration_ (h)	*t*_spinning 3 samples_ (h)
I	15	-	24	24	0.5	24	0.25	0.15
	18			24	0.5		0.25	0.15
	20		24	24	0.5		0.25	0.15
	24			24	0.5		0.25	0.15
II	15	5 wt% PVP	24	24	0.5		0.25	0.15
	18			24	0.5		0.25	0.15
	20		24	24	0.5		0.25	0.15
	24			24	0.5		0.25	0.15
III	15	5 wt% PEG400	24	24	0.5		0.25	0.15
	18			24	0.5		0.25	0.15
	20		24	24	0.5		0.25	0.15
	24			24	0.5		0.25	0.15
Time spent on 12 solutions, h			144	288	6	24	3	1.8
Total time, h			438			28.8		
